# Author response to “Stroke thrombolysis or not for an intraventricular thrombus”

**DOI:** 10.1186/s42466-023-00236-w

**Published:** 2023-03-20

**Authors:** Dimitrios Tsiptsios

**Affiliations:** Neurology Department, Democritus University of Thrace, Alexandroupolis, Greece

The aim of this letter to the editor is to clarify the comments and concerns raised by Professor Josef Finsterer and Dr Sounira Mehri with regard to our latest published manuscript in the Neurological Research and Practice Journal dealing with the safety and efficacy of intravenous thrombolysis (IVT) for acute ischemic stroke (AIS) in case of known left ventricular thrombus (LVT).

With regard to the patient’s family history, his father died at age 55 from myocardial infarction. However, no family history of hypertrophic or dilative cardiomyopathy or left ventricular hypertrabeculation is mentioned. Moreover, his individual history was not positive for arterial hypertension, heart failure, cardiac embolism, or arrhythmias. Furthermore, he took no medication regularly prior to hospitalization. Apart from that, the pre-hospital modified Rankin scale (mRS) was 0 (zero) and the two old ischemic strokes seen on the initial cerebral CT have not manifested clinically. 

During hospitalization in the coronary care unit of the nearby secondary care hospital creatine-kinase (CK) levels were 105 U/L, C-reactive protein (CRP) 4.69 mg/dl, and troponin levels were mentioned as normal. Post-thrombolysis CPK, CK-MB, CRP, and troponin levels at our tertiary referral hospital were 60 U/L, 22 U/L, 4.17 mg/dl, and 567,3 ng/ml, respectively.

Thrombolysis was performed within the 4.5 h time window. Thus, multimodal cerebral MRI to assess the relation between stroke core and penumbra was not considered necessary. 

Carotid ultrasound was normal and cardiac MRI was performed after discharge revealing no thrombus. 56% ejection fraction (EF) and left ventricular hypertrophy. CT of the aorta was not performed as, from our standpoint, the source of embolism was clear, ie LVT.

On 14-month follow-up mRS and NIHSS are both 0 (zero). The patient is currently on acetylsalicylic acid 100mgr once daily.

We agree that thrombectomy should have been preferred over thrombolysis in our patient. Unfortunately, it is not available at our hospital.

Yours Sincerely,

Sofia Kitmeridou, Dimitrios Tsiptsios, Dimos Tsalkidis, Evlampia A. Psatha, Ioannis Iliopoulos, Nikolaos Aggelousis and Konstantinos Vadikolias.

